# Skin laceration caused by a short distance shot from a pepper spray launcher: a case report

**DOI:** 10.1007/s00414-022-02936-5

**Published:** 2022-12-29

**Authors:** Anja Weber, Claudia Wöss, Beat P. Kneubuehl, Walter Rabl

**Affiliations:** 1 Institute of Legal Medicine, Medical University of Innsbruck, Müllerstraße 44, 6020 Innsbruck, Austria; 2 Institute of Forensic Medicine, Forensic Physics and Ballistics, University of Bern, Bern, Switzerland; 3 Bpk Consultancy GmbH, Thun, Switzerland

**Keywords:** Pepper spray launcher, Skin laceration, Bodily harm, JPX Jet Protector®, Ballistics

## Abstract

Pepper spray launchers are more precise and wind stable compared to conventional pepper sprays and are commonly used as a self-defensive tool. With the advanced potential, they may also harbour a greater risk for injuries, especially if they are not used within the suggested safety distance. If the shooting distance is below 1.5 m, energy densities may exceed the threshold energy density for the penetration of skin leading to skin laceration. We present a case where a man is hit by the liquid jet of a JPX Jet Protector® with an estimated shooting distance of 0.3 m. The man suffered from a bleeding skin laceration, which had to be sewed in the hospital. This case report furthermore outlines the potentially dangerous effect of pepper spray launchers and thereby their role in forensic investigations.

## Introduction

Pepper spray is typically known as conventional spray filled with a liquid formulation harbouring rather short effective ranges (1–3 m). In contrast to that, pepper spray launchers allow a more stable and targeted use of the irritant for up to 7 m of distance [1]. The irritant capsaicin (8-methyl-vanilyl-6-nonenamide) is extracted from the plant species *Capsicum* [2] and leads to activation of receptors for pain and heat of the eyes and the mucous membranes as well as increased blood flow in the affected area, but may also help to treat acute [3] or chronic pain [4]. This effect is used for the treatment of local pain for example in the back or neck area by topically applying capsaicin-containing pads [2]. Pepper sprays are used by law enforcement or for self-defence against animals and/or offenders and are generally considered harmless. Despite this, there is leading evidence that the contact with pepper spray could lead to more or less persisting injuries, especially corneal and conjunctival damage [5–9]. Generally, the ability of a projectile to penetrate the skin, causing severe injury, is influenced by the cross-section load, determined by relation of the projectile’s mass and the size of the impinging area in target direction. Among this, also the striking velocity and the material of the surface that is hit have to be considered.

The impinging energy of a projectile to the body is given in Joule (J). For penetration of the skin, an energy density of at least 0.1 J/mm^2^ and for the eyes 0.03 J/mm^2^ is needed [10, 11]. According to the manufacturer of the used launcher for this case report, the minimal distance where neither the peak energy density of the skin nor the eyes will be exceeded is determined with 1.5 m [1, 10, 12]. If the shooting distance is less, it is possible that the liquid irritant can penetrate the skin, especially if the affected body part is unclothed. To our knowledge, this is the first case reported, where the use of a pepper spray launcher led to skin laceration.

## Casuistic

**Fig. 1:**
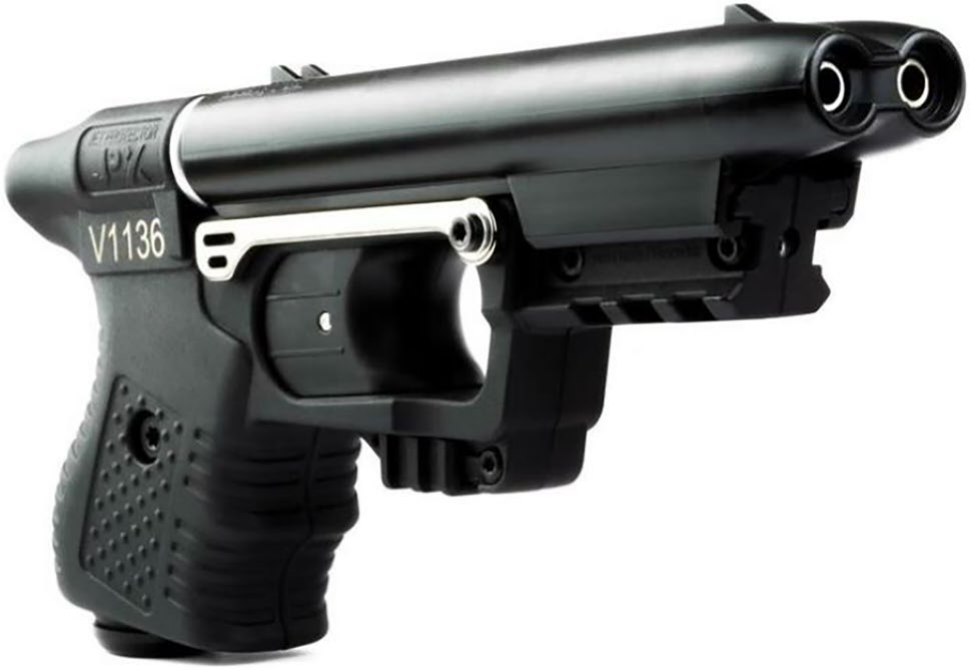
The pepper spray launcher “Jet Protector JPX®” [13]

**Fig. 2:**
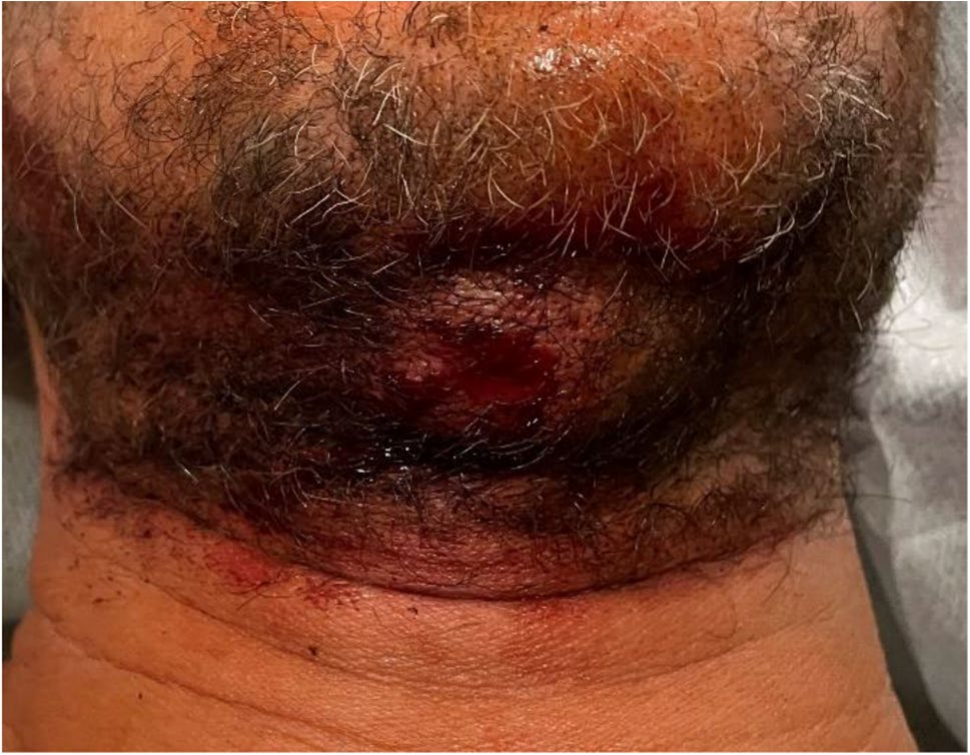
Skin wound caused by the second pepper spray shot

**Fig. 3:**
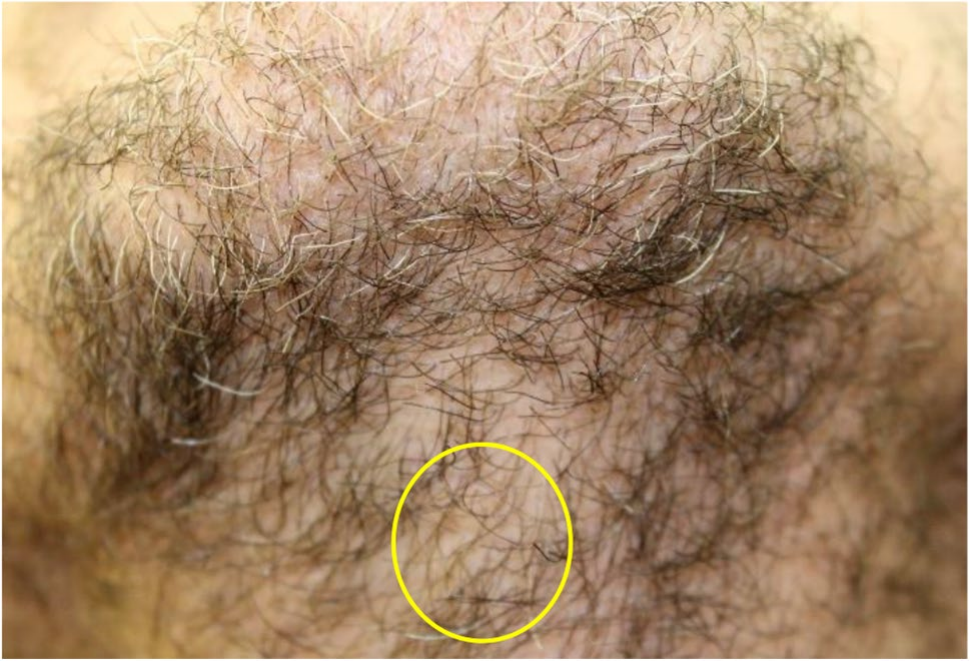
Appearance of the scar about half a year after the incidence

A dispute between two men starting in a pub escalated into a fight in the nearby parking lot. During this conflict, both men fell to the ground, and the younger man fired a pepper spray launcher twice in the direction of his opponent, a 45-year-old male. The used launcher was a Jet Protector JPX® (Fig. 1), manufactured by Piexon AG (Switzerland), which accelerates the agent by means of a pyrotechnically generated pressure for a precise shot of a bundled beam of a 10% capsaicin solution. One shot failed its target, but the other one hit the man in the front area of the neck underneath his chin and subsequently caused a skin laceration of 0.5 cm (Fig. 2), which had to be sewed in the hospital. Furthermore, first-degree burn of breast and facial skin as well as irritation of the eyes was diagnosed in the hospital. The man suffered from mild pain for about 2 more days after the incident. The wound healed smoothly with hardly any remaining scar (Fig. 3).

## Ballistic background

A liquid jet consists of a limited mass of liquid which moves at a certain velocity. The jet therefore possesses kinetic energy and also a certain energy density, which is related to the diameter of the jet. This suggests the comparison with a projectile. One major difference between a projectile and a jet is the length of the exposure time. While the impact time of a bullet lasts only fractions of milliseconds (given by the bullet length and its velocity), a jet can continue to act for several milliseconds or even hundredths of a second. This suggests that the penetration capacity of a jet is not given by the energy density, but by the energy density per unit of time, the so-called energy flux density.

In order to cause open skin wounds, jets have to produce energy flux densities which, in a very short exposure time (a matter of milliseconds), create energy densities in excess of the threshold energy density for the penetration of skin (0.1 J/mm^2^) [11].

Example: A hydraulic pipe under 10 bar pressure results in an outflow velocity of the fluid of approx. 50 m/s and an energy flux density of 0.05 J/(mm^2^⋅ms). A 10 cm long jet (exposure time 2 ms) thus already reaches the limit energy density for skin.

**Fig. 4:**
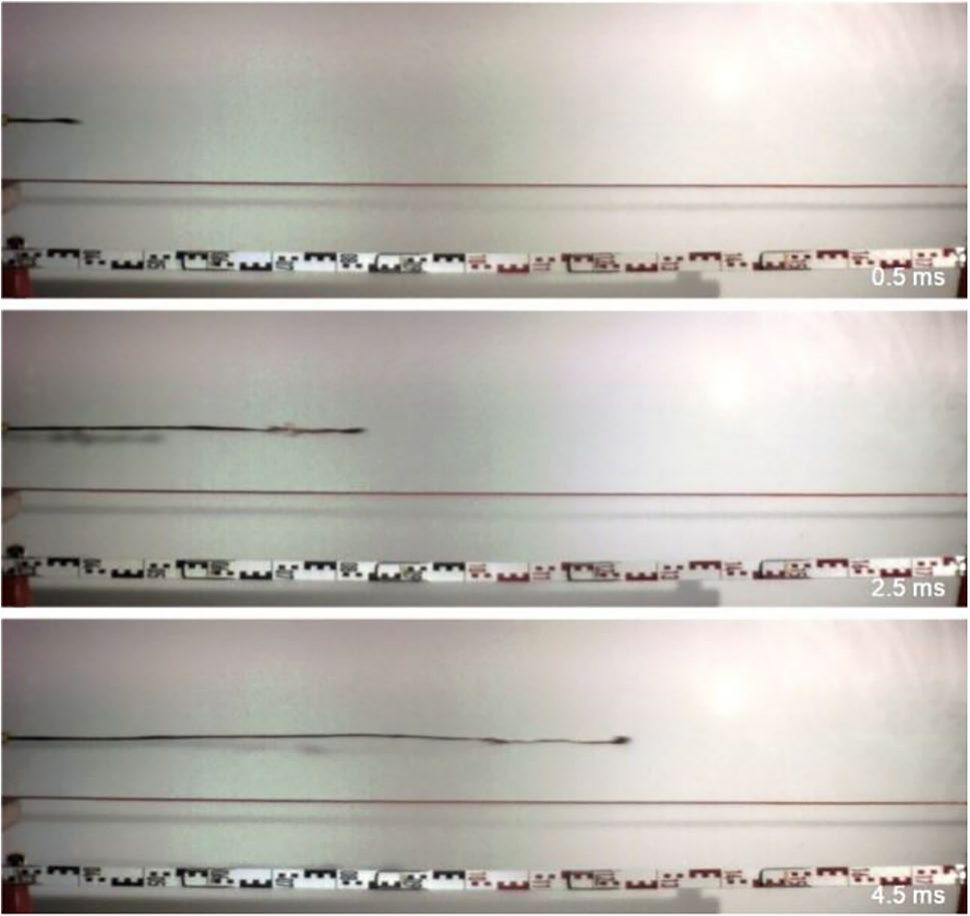
Initial dispersion of a jet from a cartridge-powered irritant pistol. Muzzle on the left side (extract from a high-speed video performed by the group of forensic physics/ballistics at the ballistic laboratory of the legal medicine Bern under supervision of Beat P. Kneubuehl)

Immediately after leaving the nozzle, the tip of the jet of the JPX reaches velocities of about 200 m/s (as established by high-speed video; see Fig. 4).

However, it decelerates rapidly thereafter, falling to around 50 m/s after 4 m. Up to 1.5 m after the muzzle, the JPX is classified as dangerous. As mentioned above, the ability of such a jet to cause injury depends on its energy flux density. The jet opening angle is thus also relevant, as it determines the impact area.

In the present case, a shooting distance of about 0.3 m is to be assumed. At this distance, a jet velocity of approx. 180 m/s [10, 11] can be expected, with the entire mass of the jet (approx. 9 g) hitting the target. This results in an impact energy of 146 J. With a jet opening angle of 3–4°, a beam diameter of 30–40 mm will result at 0.3 m. This results in an energy density of 0.11–0.2 J/mm^2^. With these values of energy and energy density, it is obviously possible for the jet to produce first a contusion and then an open injury.

## Discussion

Pepper sprays as handheld pressurized dispensers are widely used for self-defence, but are gaining more and more attention in the field of forensics as they may be involved in several kinds of criminal activities. Borusiewicz et al., for example, tried to distinguish the type of pepper spray by the composition of capsaicinoids in order to find ways to compare the used pepper spray with the one that was found with a potential suspect. They could show that oleoresin capsicum formulations can be distinguished even if they originate from the same manufacturer as long as a different batch has been used [14]. The further developed pepper spray launchers, like the JPX Jet Protector that has been used in the presented case, show increased effective ranges for up to 7 m and can shoot 2 doses of 10% oleoresin capsicum with a muzzle speed about 200 m/s [1]. In contrast to the conventional pepper sprays, which show typical effective ranges of up to 3 m, these launchers are also more precise, wind stable, and some are even featured with a laser to better target the direction [15]. If these launchers are used from short distance, the beam that hits the skin is much more powerful compared to conventional pepper sprays. Kniestedt et al. report about two cases where persisting injury of the eyes occurred after an accidental self-inflicted shot with capsaicin using a Jet Protector Guardian Angel®. The severity of the injury was attributed to the impact energy of the jet of the used launcher, leading to penetration of the corneal epithelia, which is not possible using a conventional spray without pyrotechnic ignition [16]. In Austria, pepper sprays are classified as weapons according to §1 WaffG and §3 StGB and are therefore not allowed to be held by underage persons and must only be used to act in self-defence [17]. This could be difficult if using a pepper spray launcher with effective ranges up to 7 m as one can shoot from a large distance before a person is even close enough to harm. As we could show for the reported case, a shot from close range with these kinds of launchers may cause injuries that are hard to estimate, depending on the actual distance and body part that is hit. Therefore, the manufacturer outlines a safety distance of 1.5 m, which has been proven by measurement of energy and energy density [12]. Despite the legal position in Austria, where pepper spray pistoles are allowed to be purchased and carried along for individuals over 18 years of age [18], in Switzerland, the JPX Jet Protector® is classified as weapon within the meaning of imitation weapons, arms used as warning devices, and soft air weapons according to Art.4 Abs. 1 Bst. g WG. The purchase is allowed without report to the regional gun office. Nevertheless, a legal permission is needed if a person wants to carry the JPX Jet Protector® along. For pepper spray pistoles using a laser to aim, special permission is needed [19, 20].

Despite the declaration as a rather harmless self-defensive tool, there have already been deaths reported that were associated with the use of pepper sprays. Granfield et al. report about several in-custody cases where pepper spray was a contributing cause of death in 2 of 63 fatalities. In one case, signs of pre-existing asthma have been found, which have potentially been enforced by the inhalation of pepper spray agents. The other case showed bronchial damage, which probably enhanced the chance of bronchial spasms when inhaling pepper spray. In the other 61 cases, death occurred due to drug intake, disease, positional asphyxia, or a combination of those [21]. This study outlines that, especially in case of pre-existing illness, pepper sprays can be considered as potentially dangerous weapons. For our reported case with an estimated shooting distance of 0.3 m, we could show that pepper spray launchers may cause skin laceration, as this distance results in an energy density of 0.11–0.2 J/mm^2^ and is therefore exceeding the energy density for the penetration of skin, which further emphasizes the predetermined safety distance of 1.5 m.

## Data Availability

Data sharing not applicable to this article as no datasets were generated or analysed during the current study.
